# Sleep-Dependent Anomalous Cortical Information Interaction in Patients With Depression

**DOI:** 10.3389/fnins.2021.736426

**Published:** 2022-01-06

**Authors:** Jiakai Lian, Yuxi Luo, Minglong Zheng, Jiaxi Zhang, Jiuxing Liang, Jinfeng Wen, Xinwen Guo

**Affiliations:** ^1^School of Biomedical Engineering, Sun Yat-sen University, Guangzhou, China; ^2^Guangdong Provincial Key Laboratory of Sensing Technology and Biomedical Instruments, Sun Yat-sen University, Guangzhou, China; ^3^Institute for Brain Research and Rehabilitation, South China Normal University, Guangzhou, China; ^4^Department of Psychology, Guangdong 999 Brain Hospital, Guangzhou, China

**Keywords:** sleep, depression, electroencephalography, effective connectivity, delay symbolic phase transfer entropy

## Abstract

Depression is a prevalent mental illness with high morbidity and is considered the main cause of disability worldwide. Brain activity while sleeping is reported to be affected by such mental illness. To explore the change of cortical information flow during sleep in depressed patients, a delay symbolic phase transfer entropy of scalp electroencephalography signals was used to measure effective connectivity between cortical regions in various frequency bands and sleep stages. The patient group and the control group shared similar patterns of information flow between channels during sleep. Obvious information flows to the left hemisphere and to the anterior cortex were found. Moreover, the occiput tended to be the information driver, whereas the frontal regions played the role of the receiver, and the right hemispheric regions showed a stronger information drive than the left ones. Compared with healthy controls, such directional tendencies in information flow and the definiteness of role division in cortical regions were both weakened in patients in most frequency bands and sleep stages, but the beta band during the N1 stage was an exception. The computable sleep-dependent cortical interaction may provide clues to characterize cortical abnormalities in depressed patients and should be helpful for the diagnosis of depression.

## Introduction

Depression is a prevalent mental illness with high morbidity and encompasses abnormal performances such as anhedonia, low self-esteem, and even self-mutilation, which is considered the main cause of disability worldwide (World Health Organization [WHO], 2019). Cumulative neuroscience research on resting state and various cognitive tasks have suggested that the dysregulated cortical and subcortical functional network, which is considered to affect brain function integration and information interaction, was found in depressed patients (Furman et al., 2011; de Kwaasteniet et al., 2013; Ma et al., 2021). Moreover, brain activities while sleeping were also reported to be affected by such mental illness (Steiger and Pawlowski, 2019).

Sleep is significant for the regulation of brain function, including the adjustment of cerebral cortex activity to preserve the homeostasis of the functional network (Tononi and Cirelli, 2006; Krueger et al., 2016). During sleep, brain’s response to external stimuli is weakened and, thus, specific spontaneous pathological information of neurological or mental diseases can be observed (Berry, 2012). The majority of studies regarding depression during sleep have investigated the polysomnographic alterations and some typical differences have been found: the longer sleep latency, the decreased sleep efficiency, prolonged first rapid eye movement (REM) stage, and reduced slow wave sleep (Tsuno et al., 2005; Sculthorpe and Douglass, 2010; Murphy and Peterson, 2015). However, scant research worked on exploring sleep functional network of depressed patients. Synchronous likelihood was utilized on sleep electroencephalography (EEG) signals to find that lower mean level of global synchronization was present in depressed patients (Leistedt et al., 2009). According to linear Granger causal analysis, small-world network organization in patients with depression was altered during REM sleep (Hein et al., 2019). Moreover, Zhang B. T. et al. (2020) used connectivity metrics derived from two sleep EEG channels to obtain sound results in depression screening. In light of the existing research background, the special relationship between depression and sleep should not be overlooked. Further investigation on the sleep cerebral functional network of depression may help us more comprehensively understand the pathological mechanism of depression.

In virtue of noninvasive high time resolution, long-range timely recording, and relatively low physiological load, EEG is deemed as an ideal tool for studying cerebral activity during sleep. The current functional network analyses based on cortical EEG include functional connectivity (FC) and effective connectivity (EC), which are both based on the functional properties of the various cortical regions (Friston, 2011; Stam, 2014). FC represents the temporal correlations that imply direct or indirect interactions between brain regions (Cai et al., 2018), whereas EC refers to a kind of directional causal influence that neural masses exert upon each other, which should be more comprehensive to illuminate the cerebral activity (Valdes-Sosa et al., 2011). Currently, informatics methods were widely applied to EC analysis for further directional brain network investigation, and based on which, transfer entropy (TE) was proposed as an EC measure to study the information flow in the cortical network (Vicente et al., 2011). Since the cortical EEG is easily affected by the volume conduction (He et al., 2019), in recent years, constantly improved algorithms have been proposed to reduce this effect on scalp estimates of EC and improve the reliability and stability of the calculations, and related applications on research unveiled anomalous cerebral information interaction in depressed patients. Cukic et al. (2020) applied transfer entropy on resting EEG and found that the frontal, parietal, and temporal lobes of patients are relatively isolated. A more randomized brain network structure was found in patients in accordance with phase transfer entropy analysis (Hasanzadeh et al., 2020). Zhang Y. et al. (2020) used multivariate symbolic transfer entropy to find that the connection strength of patients between the left occipital area and the frontal lobe area under the stimulation of positive and neutral emotional pictures was significantly different from that of healthy controls. Recently, delay symbolic phase transfer entropy (dSPTE), a new extension of TE incorporating the advantages of phase information analysis and symbolic scheme, was proposed to quantify brain activity EC, which has better noise robustness and more accurate identification of EC (Wang and Chen, 2020). With the advantages of nice stability and accuracy, dSPTE was applied in this study to investigate the functional interactive network in depressed patients during sleep.

Studies have implied the cerebral functional asymmetries in depressed patients, and the anomalous functional network may lead to the aberrant symptoms such as abnormal information processing and excessive rumination (Rotenberg, 2008; Bruder et al., 2012). However, insufficient investigations committed to reveal and characterize the asymmetries of cortical information flow in depressed patients during sleep, the topic of which was considered to provide valuable information for the abnormal cerebral function in patients. Moreover, previous studies have tried to explore different inter-regional features to quantify different patterns of cortical information flow, such as left–right index and anterior–posterior rate (Zhou et al., 2020; Ekhlasi et al., 2021; Pan et al., 2021). To discover the difference in sleep-dependent information flow patterns in patients, analysis on various EC asymmetry patterns was included in this research.

In this study, we tried to explore the changes in the interactive functional network in patients with depression during sleep. The information transfer between cortical regions and two information flow patterns (left–right pattern, posterior–anterior pattern) in different sub-bands and sleep stages were considered to analyze the differences between patients with depression and healthy controls. We expect to provide new insights into the understanding of pathological mechanisms in depression.

## Materials and Methods

### Participants

Twenty-five patients with depression and twenty-six age-matched healthy controls were enrolled in our study, and the clinical characteristics of participants are listed in Table 1. Patients from Guangdong 999 Brain Hospital were diagnosed by two experienced psychiatrists based on criteria of Diagnostic and Statistical Manual of Mental Disorders, Fourth Edition (DSM-IV). Patients with depression were also assessed by the Hamilton Depression Scale (HAMD) and the Self-Rating Depression Scale (SDS). The exclusion criteria for patients with depression included the presence of drug abuse, suicide risk, pregnancy, present or history of head injuries, seizures, or epilepsy. Healthy participants were recruited from Sun Yat-sen University and had no history of nerve damage, no family history of psychiatric disorders, no history of sleep disorders, and no history of drug or alcohol abuse. Participants in the experiment were not interfered by medications under the judgment of an experienced clinical psychiatrist, and had not experienced sleep deprivation and other disturbances. This study had the approval of the Ethics Committee of Guangdong 999 Brain Hospital (approval number: 2020-010-059). All procedures performed in this study were in accordance with the 1964 Helsinki declaration and its later amendments. All participants voluntarily signed an informed consent form before the experiment and were appropriately remunerated after the experiment.

**TABLE 1:** Demographic and clinical data for participants.

Variables	Healthy controls	Depressed patients	*p*-value
Age (years)	20 ± 1.50	21.6 ± 7.04	0.947
Gender, male/female	13/13	14/11	0.668
HAMD score	2.08 ± 1.57	25.5 ± 6.28	<0.001
SDS score	40.92 ± 6.99	67.3 ± 9.91	<0.001
Total Analysis Time (min)	458.13 ± 96.84	583.75 ± 55.17	/
NREM1 Time (min)	25.17 ± 12.45	22.89 ± 15.58	0.205
NREM2 Time (min)	181.71 ± 41.39	253.81 ± 82.91	0.001
NREM3 Time (min)	141.21 ± 37.31	146.45 ± 55.56	0.445
REM Time (min)	82.03 ± 22.17	103.22 ± 50.08	0.220
Total Sleep Time (min)	429.40 ± 41.59	526.33 ± 74.11	/

*All data are presented as mean ± standard deviation, except for gender. HAMD, Hamilton Depression Scale; SDS, Self-rating Depression Scale. The comparison of gender was assessed with chi-squared test and the other comparisons were assessed using the Mann–Whitney U test.*

### Polysomnography

All participants underwent overnight polysomnography (PSG) examination using a Compumedics Profusion EEG recording system with Neuvo amplifier, and the recording lasted for 9–10 h. Six scalp EEG channels (F3/M2, F4/M1, C3/M2, C4/M1, O1/M2, and O2/M1) following the 10–20 system were selected for the study at a sampling rate of 500 Hz. The reference electrodes (M1 and M2) were placed on contralateral auricle and a ground electrode was on Fpz according to the recommendation of the American Academy of Sleep Medicine (AASM) criteria (Berry et al., 2017). Moreover, electrooculography, electrocardiography, electromyography, oral and nasal respiratory airflow, chest and abdomen breathing movement, blood oxygen saturation, snoring, leg movement, and body position were also recorded. Sleep stages (REM, N1, N2, N3, and Wake) were then scored by two experienced sleep technicians according to the AASM criteria.

### Electroencephalography Signal Pre-processing

The EEG recordings were divided into 30-s epochs for sleep scoring. Segments with obvious artifacts were excluded by visual inspection. Finally, 18,422 segments from depressed patients (694 W epochs, 4,571 R epochs, 421 N1 epochs, 6,973 N2 epochs, and 5,763 N3 epochs) and 18,691 segments from healthy controls (563 W epochs, 2,997 R epochs, 540 N1 epochs, 7,737 N2 epochs, and 6,854 N3 epochs) were obtained. Then, middle 10-s segments from these epochs were extracted for analysis, and a fourth-order zero phase shift Butterworth band-pass filter (0.5–60 Hz) was used to denoise the raw EEG signals.

### Directionality Analysis

To determine the directed information flow between cortical regions, dSPTE was estimated based on EEG signals. Under the framework of directional dynamic analysis, TE evaluates the degree of influence of the driving time series on the target one (Schreiber, 2000). Suppose a causal relation between source signal X and target signal Y, uncertainty of the target signal prediction would be reduced when adding both its own past information and that of the source signal:


$$TE_{X\to Y}=\sum P\,\left(Y_{t},Y_{t-\delta },X_{t-\delta }\right)\,\text{ log }\,\left(\frac{p\,\left(Y_{t}|Y_{t-\delta },X_{t-\delta }\right)}{p\,\left(Y_{t}|Y_{t-\delta }\right)}\right)$$

Developed from TE, the dSPTE has better noise robustness and can correctly identify the EC, and its calculation procedure contains phase information extraction, symbolic process, and true delay search. For phase information extraction, a combined Morlet wavelet was used to obtain the instantaneous phase (Liao et al., 2019). The definition of a single Morlet complex wavelet is:


$$\sigma \,\left(t\right)=\frac{1}{\sqrt{\pi \,f_{b}}}\,e^{2\,i\,\pi \,f_{c}\,t}\,e^{-\frac{t^{2}}{f_{b}}}$$

where f_c_ is the center frequency of the wavelet and f_b_ is the bandwidth parameter. Then, Morlet complex wavelets with different center frequencies f_n_ were superimposed to obtain the combined Morlet wavelet, and the f_n_ can be expressed as:


$$f_{n}=f_{L}+n\times △\,f,n=0\,\ldots \,N-1$$

where △*f* was the center frequency spacing of wavelet, and *f*_*L*_ and N were the central frequency of the first wavelet and the number of wavelets, respectively. The combined Morlet wavelet is defined as:


$$\Psi_{c}\,\left(t\right)=\frac{1}{C}\,\sum_{n=0}^{N-1}\sigma_{f_{n}}\,\left(t\right)=\frac{1}{C\,\sqrt{\pi \,f_{b}}}\,e^{-\frac{t^{2}}{f_{b}}}\,\sum_{n=0}^{N-1}e^{2\,i\,\pi \,f_{n}\,t}$$

where C is the correction coefficient that makes the amplitude-frequency characteristic passband of the combined wavelet equal to 1. For the EEG signal S(t), its phase information φ (τ) can be obtained after convoluting with the combined Morlet wavelet. The wavelet coefficient at time τ is defined as:


$$W_{S}\,\left(\tau \right)=\int_{-\infty }^{\infty }S\,\left(t\right)\,\Psi_{C}^{*}\,\left(t-\tau \right)\,dt=A\,\left(\tau \right)\,e^{i\,\varphi \,\left(\tau \right)}$$

In this study, the f_b_ = 2 and △*f* = 0.05, and the parameters *f*_*L*_ and N were *f*_*L*_ = 0.5 and *N* = 70 for delta (0.5–4 Hz), *f*_*L*_ = 4 and *N* = 80 for theta (4–8 Hz), *f*_*L*_ = 8 and *N* = 80 for alpha (8–13 Hz), and *f*_*L*_ = 12 and *N* = 400 for beta (13–32 Hz). Then, we performed a symbolic process based on permutation entropy (Staniek and Lehnertz, 2008), assuming $\theta_{t}^{x}$ is the phase series extracted from random time series x(t). To better capture the underlying dynamics of the series, the past space state is reconstructed through a time embedding method, so the space state of $\theta_{t}^{x}$ is approximated as:


$$\theta_{t}^{x\,d}=\left[\theta^{x}\,\left(t\right),\theta^{x}\,\left(t-l\right),\ldots \,\theta^{x}\,\left(t-\left(m-1\right)\,l\right)\right]$$

where *m* and *l* are the embedding dimension and delay, respectively. Then, the values are arranged in an ascending order [θ*^x^* (*t* − (*j*_1_ − 1) *l*) ≤ θ*^x^* (*t* − (*j*_2_ − 1) *l*) ≤ … ≤ θ*^x^* (*t* − (*j*_*m*_ − 1) *l*)], and the symbol is defined as $S_{t}^{\theta^{x}}=\left[j_{1},j_{2},\ldots ,j_{m}\right]$. In this study, *m* = 5 and *l* = 62, 31, 19, and 7 were selected for the delta, theta, alpha, and beta frequency bands, respectively (Li et al., 2017; Zubler et al., 2018). For two discrete time series X and Y, the information transfer from X to Y will be the maximal under the real delay (Wang and Chen, 2020). To obtain the optimal dSPTE, the interaction lag parameter μ between the driving and driven systems was set from 1 to 15 to find the optimal dSPTE. The dSPTE is expressed as:


$$d\,S\,P\,T\,E_{X\to Y}=\sum p\,\left(S_{t}^{\theta^{y}},S_{t-1}^{\theta^{y}},S_{t-\mu }^{\theta^{x}}\right)\,l\,o\,g\,\frac{p\,\left(S_{t}^{\theta^{y}}|S_{t-1}^{\theta^{y}},S_{t-\mu }^{\theta^{x}}\right)}{p\,\left(S_{t}^{\theta^{y}}|S_{t-1}^{\theta^{y}}\right)}$$

### Inter-Regional Effective Connectivity Pattern

Based on the dSPTE, left–right index (LR) and anterior–posterior ratio (AP) were introduced to assess the different information flow patterns in the cortical regions. To obtain these indices, normalized dSPTE was used:


$$n\,d\,S\,P\,T\,E_{x\,y}=\frac{d\,S\,P\,T\,E_{x\to y}}{d\,S\,P\,T\,E_{x\to y}+d\,S\,P\,T\,E_{y\to x}}$$

where *ndSPTE*_*xy*_ ranging from 0.5 to 1 means the information flows preferentially from X to Y, and *ndSPTE*_*xy*_ ranging from 0 to 0.5 means the reverse situation. For each EEG channel, we averaged its *ndSPTE*_*xy*_ with all the other channels to get its regional *ndSPTE* values, which indicated whether the information transmission role of a cortical area was a driver (0.5 < *ndSPTE* < 1) or a receiver (0 < *ndSPTE* < 0.5). LR represented the relative transmission direction of information and the degree of difference in information exchange between the left and right hemisphere (Zubler et al., 2018), which was defined as follows:


$$\text{LR}={\left\{\lambda \,\frac{n\,d\,S\,P\,T\,E_{L\,R}-n\,d\,S\,P\,T\,E_{R\,L}}{n\,d\,S\,P\,T\,E_{L\,R}+n\,d\,S\,P\,T\,E_{R\,L}}\right\}}_{a\,v\,e\,r\,a\,g\,e}$$

where λ = 1, and *ndSPTE*_*LR*_ (*ndSPTE*_*RL*_) was the normalized dSPTE from left to right (right to left), and was calculated with the electrode pairs of left and right hemispheres, including F3-F4, C3-C4, and O1-O2. LR > 0 indicated the left-to-right hemispheric information flow and *vice versa*. The closer the LR value was to 0, the smaller the difference between inter-hemispheric information flows.

Anterior–posterior ratio (AP) was defined to assess the anterior-to-posterior pattern of the information flow (Numan et al., 2017):


$$\text{AP}=\frac{{\left\{n\,d\,S\,P\,T\,E_{A\,P}\right\}}_{a\,v\,e\,r\,a\,g\,e}}{{\left\{n\,d\,S\,P\,T\,E_{P\,A}\right\}}_{a\,v\,e\,r\,a\,g\,e}}$$

where *ndSPTE*_*AP*_ (*ndSPTE*_*PA*_) was the normalized dSPTE from anterior to posterior (posterior to anterior), and was calculated with the electrode pairs of anterior and posterior regions, including F3-C3, F3-O1, C3-O1, F4-C4, F4-O2, and C4-O2. The information flow direction is anterior-to-posterior, AP > 1, whereas the opposite direction retrieves 0 < AP < 1, and a balanced direction retrieves AP = 1. The closer the AP value was to 1, the smaller the difference between anterior and posterior information flows.

### Statistical Analysis

Statistical tests were conducted to address discrepancies in the cortical interactive network between the healthy controls and depression groups during sleep in various frequency bands and researched sleep stages. Since dSPTEs, LRs, and PAs did not satisfy the normal distribution or the variance homogeneity test, a non-parametric test (Mann–Whitney *U* test) was used to test the significant difference (*p* < 0.05 was considered statistically significant). Furthermore, the Friedman test was utilized to test the null hypothesis that the features of EC between different sleep stages were the same, and the Bonferroni correction was conducted to access the stage-dependent significant differences if the Friedman test showed a significant difference (*p* < 0.05). All analyses were performed using IBM statistical software version 22.0.

## Results

### Information Transfer Across Cortical Regions

**FIGURE 1:**
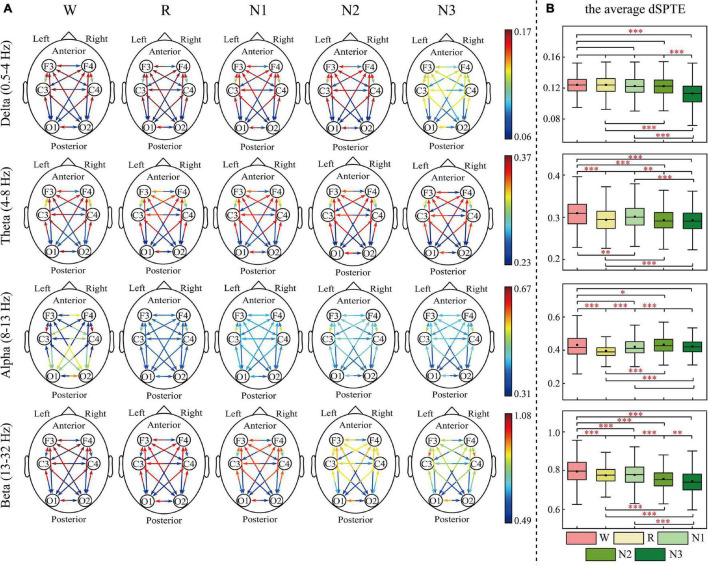
dSPTE of healthy controls during five stages in four frequency bands. **(A)** Overall dSPTE across cortical regions. Each monodirectional arrow indicates the directed influence of one cortical region to another. The warmer the color of the line with the arrow, the stronger the intensity of EC. **(B)** The average dSPTE across six electrodes in five stages. Asterisks denote the significant difference between two stages. *p < 0.05, **p < 0.005, and ***p < 0.001 (Bonferroni correction).

**FIGURE 2:**
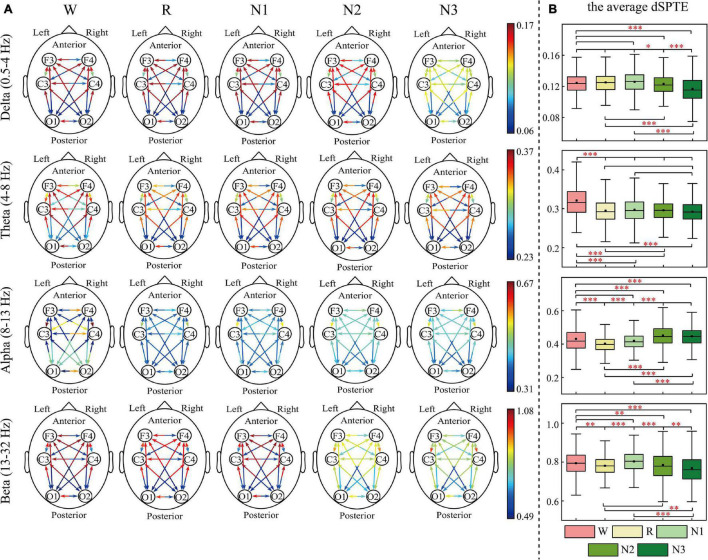
dSPTE of depressed patients during five stages in four frequency bands. **(A)** Overall dSPTE across cortical regions. Each monodirectional arrow indicates the directed influence of one cortical region to another. The warmer the color of the line with the arrow, the stronger the intensity of EC. **(B)** The average dSPTE across six electrodes in five stages. Asterisks denote the significant difference between two stages. *p < 0.05, **p < 0.005, and ***p < 0.001 (Bonferroni correction).

The EC networks during sleep were represented by the dSPTE between EEG channels. Figures 1, 2 show the EC network of the healthy controls and that of the depressed patients, respectively. Through the color of the arrows, the advantage transfer directions between channels can be obtained. As shown in Figures 1A, 2A, in delta, theta, and beta bands, most information flow between the two channels has an obvious advantage direction, which means the information transfer in one direction was obviously larger than the other.

In terms of the inter-hemispheric information flow, except for the alpha band, the information transfer into the left hemisphere was larger than the other direction. We also observed that the information transition of occipital regions had a uniform directional tendency, except for the alpha band, where the information transferred from occipital regions was larger than the opposite direction.

The information transition of frontal regions also indicated directional tendency, but the results depended on frequency bands and stages. For the low-frequency bands, delta and theta, the information transfer into the frontal regions was larger, but in delta during N3 sleep, this tendency slightly weakened. The information transferred from occipital regions to frontal regions was slightly larger than the other direction during sleep in the alpha band. For the beta band, such tendency differed during various sleep stages. During Wake, R, and N1, the information into frontal regions was larger, but during N3 stages, information transfer from the frontal regions to central regions was larger than the other direction. We also calculated the average dSPTE in the whole cortical network in different sleep stages for reference (Figures 1B, 2B; the specific values were listed in Supplementary Table 1).

### Differences in Information Transfer Across Cortical Regions Between Patients With Depression and Healthy Controls

**FIGURE 3:**
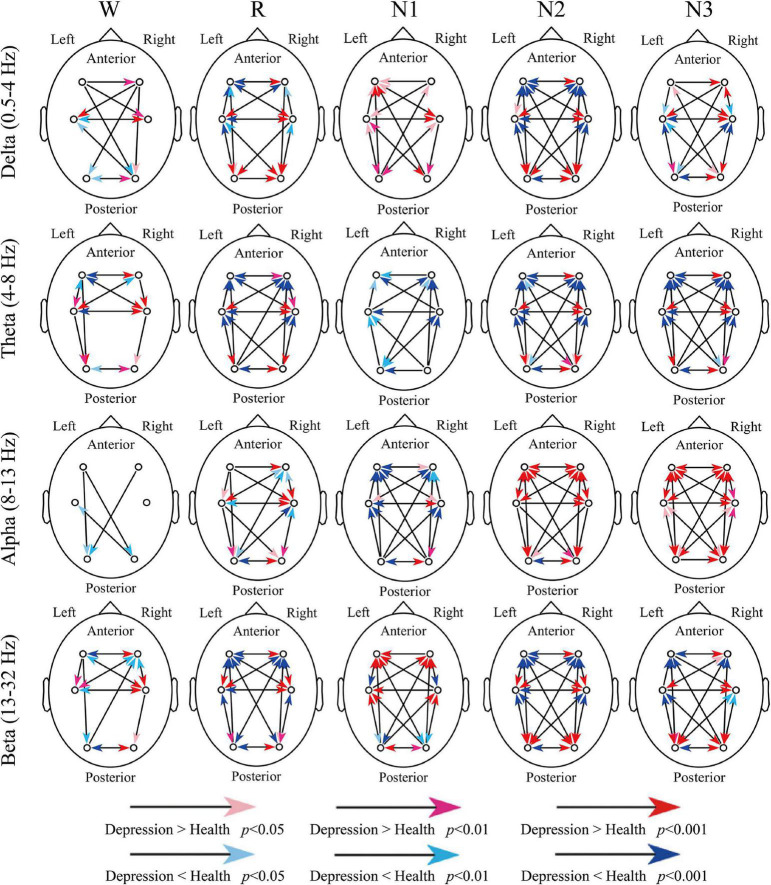
Differences in dSPTE between depressed patients and healthy controls. In each subgraph, the red arrows indicate the significantly stronger flow in depressed patients compared with healthy controls, whereas the blue arrows indicate the opposite condition.

Figure 3 indicates all the significant difference between groups. For delta and theta bands, except for a few compression results, such as the inter-hemispheric information transfer between frontal and occipital regions (O2-F3 and O1-F4), the significant difference weakened the advantage information transfer direction in depressed patients. For example, the advantage direction between F3 and F4 was F4 to F3, but the depressed patients indicated a stronger information transfer from F3 to F4 or weaker transfer from F4 to F3.

For the alpha band, its advantage direction during sleep was not so obvious as other bands. During R and N1 stages, the anterior-to-posterior information flow increased and the opposite information flow decreased, and the information transfer from the left hemisphere increased in patients. However, during N2 and N3 stages, it seemed that the information transfers in the whole cortical network increased in patients, except for O1 to O2. For the beta band, during Wake, R, N2, and N3 stages, the advantage direction weakened in patients like the results of delta and theta bands. However, during N1, these significant differences further enhanced the directional tendency in patients, which may be the result we should notice.

### Anterior-to-Posterior Pattern and Left-to-Right Pattern of Information Flow

**FIGURE 4:**
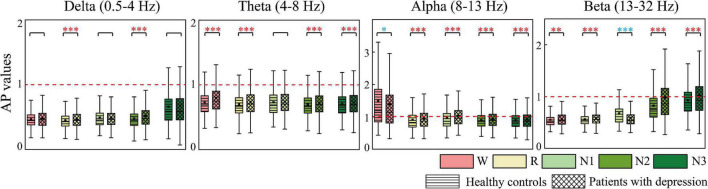
Differences in AP between groups. Red asterisks indicated that the AP of depressed patients significantly increases compared to the controls, whereas the opposite is observed in the other case (blue). *p < 0.05, **p < 0.005, and ***p < 0.001. The red dotted line is the baseline of AP (AP = 1).

**FIGURE 5:**
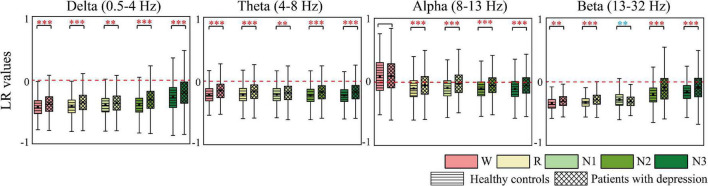
Differences in LR between groups. Red asterisks indicated that the LR of depressed patients significantly increases compared to the controls, whereas the opposite is observed in the other case (blue). * *p* < 0.05, **p < 0.005, and ***p < 0.001. The red dotted line is the baseline of LR (LR = 0).

AP and LR were constructed to summarize the information flow tendency between hemispheres and the anterior–posterior pattern of EC network, the group differences of which are shown in Figures 4, 5, respectively. Supplementary Figure 1 in the Supplementary Material shows the difference in AP and LR values across different sleep stages; the specific values of AP and LR are listed in Supplementary Table 2. Except for the alpha band, the other three bands had an obvious hemispheric bias (LR < 0). The overall information flow was from the right to the left cerebral hemispheres, and the information flow tendency was from posterior to anterior (AP < 1). During N3 in delta and during N2 and N3 stages in beta, this hemispheric bias and posterior-to-anterior tendency weakened. During wake in alpha band, this hemispheric bias and anterior-posterior information flow pattern were reversed. The above hemispheric bias and posterior-to-anterior tendency both weakened in patients; the LRs were closer to 0 and APs were closer to 1 in the patient group. With the following exceptions, during the N1 stage, the AP and LR of beta in patients further decreased.

### Difference in Regional Information Between Patients With Depression and Healthy Controls

**FIGURE 6:**
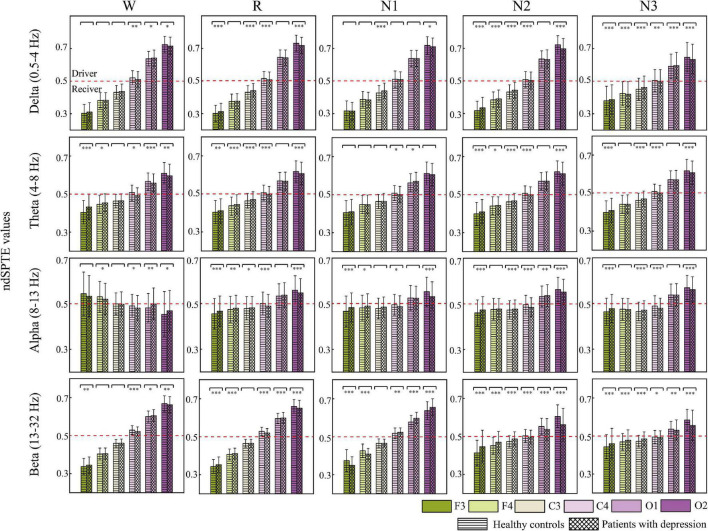
The ndSPTE for each cortical area. The regional ndSPTE represents the information transmission ability of the corresponding cortical area relative to the whole brain. In each subgraph, twelve stacked bars with error bars are included, and six colors represent six cortical locations, and two textures represent two groups. Asterisks denote the significant difference between two groups. *p < 0.05, **p < 0.005, and ***p < 0.001. The red dotted line is the baseline (ndSPTE = 0.5) to distinguish the role of cortical areas in information transmission.

Figure 6 indicates the information transition roles of various cortical regions. Except for the converse results in the wake stage of the alpha frequency band, in most cases, the occipital areas tended to be the sender, whereas the frontal areas were the information receivers. Besides, the right hemisphere showed a stronger information drive than the left hemisphere. These information transition roles slightly weakened in the alpha band during sleep stages and in beta bands during N2 and N3 stages. For delta, theta, and beta bands, the order from receiver to driver was basically as follows: F3, F4, C3, C4, O1, and O2. C4 was the closest to the balance of information sending and receiving (*ndSPTE* = 0.5). Both groups had the results above.

In regard to the difference between groups, except in beta during N1, nearly all the significant differences made the *ndSPTE* closer to 0.5 in patients. The *ndSPTE* increased in frontal regions and decreased in occipital regions, which meant that the definiteness of role division in cortical regions was weakened in depressed patients. However, decreased *ndSPTE* in frontal regions and increased *ndSPTE* in occipital regions were found in beta during N1, which was an exception.

## Discussion

Delay symbolic phase transfer entropy was used to estimate the cortical EC network in patients with depression during sleep. Several features such as LR, AP, and regional *ndSPTE* were constructed to evaluate the information directions of inter-hemisphere, anterior–posterior, and the roles of information transition of various cortical regions, respectively. We observe obvious information flows to the left hemisphere and to the anterior cortex. For regions, the occiput tended to be the information driver, whereas the frontal regions played the role of the receiver. Such directional tendencies in information flow and the definiteness of role division in cortical regions were both weakened in patients.

Compared with previous studies (Zhou et al., 2020; Pan et al., 2021), our method provided detailed differences in information flow between brain regions, and clearly characterized the role of regions in information transmission (as a receiver or driver). It has been reported that the occipital region and the parietal region are considered to be related to visual information (Heo et al., 2018) and somatosensory information (Fogassi and Luppino, 2005), respectively, while the frontal area has a bearing on senior cognitive functions, emotions, and information integration functions (Fogassi and Luppino, 2005; Alvarez and Emory, 2006). Previous research indicated that the top-down information interaction during sleep was significant for memory consolidation (Axmacher et al., 2009; Miyamoto et al., 2016). The strong forward information flow and the difference in the role of anterior–posterior information communication that we found may reflect the integration and reprocessing of information from the episodic memory during sleep (De Gennaro et al., 2004).

Thanks to the high resolution of dSPTE for information transmission evaluation, we found that the difference in the regional role and in information flow of patients was weaker during sleep compared with healthy controls. Functional research implied that impaired bottom-up limbic cortex regulation led to abnormal mood regulation in patients with depression (Ochsner et al., 2009; Ramasubbu et al., 2014), which may also suggest such abnormal anterior–posterior information interaction in patients. A previous study showed that the information in visual working memory was presented in the occipital, parietal, and frontal cortex (Yu and Shim, 2017). In addition, a physiological study found that increased microRNA-132 levels, which were widely reported in patients with depression, were associated with impaired visual memory (Liu et al., 2016). The anomalous occipital–parietal–frontal information transfer we found may reflect the abnormality of the patient’s visual information pathway.

Similarly, such reduction in the difference in information flow was also found between the left and right hemispheres in patients. Studies found that the right hemisphere was regarded as having a relative advantage during sleep, playing a function of vigilance and control of external information (Casagrande and Bertini, 2008b,a). The strong information flow to the left presented in our results may reflect this right hemisphere superiority. Most depressed patients have sleep disorders, such as difficulty in falling asleep, unsustainable sleep, and getting up early (Weaver et al., 2018). The weakened role difference between the left and right regions in patients may affect the brain’s vigilant and control function of external information during sleep, making it hard for the brain to maintain sleep homeostasis, which may be one of the reasons for sleep disorders in patients with depression.

Different brain regions play their own functions in various functional collaborations (Genon et al., 2018), and the clear distinction between regional roles presented in our results confirms this to a certain extent. Previous studies on sleep brain dynamics found that different brain regions may fall asleep at different speeds and exhibit different sleep intensities, which may reflect the regional function differences in sleep regulation and indicate that the process of sleep is neither spatially nor temporally a uniform state (Vecchio et al., 2017; Fernandez Guerrero and Achermann, 2019). However, we found that the information communication roles of different brain regions in patients tend to be blurred during sleep. Such blurring may reflect the abnormal brain function coordination, which provides possible reasons for the impairments of the ability to process information, emotional regulation, and sleep quality in depressed patients.

In addition, the abnormal change of information flow in N1 of the beta band was found in depressed patients, which may be an important indicator and require further research to investigate.

The present study still has some limitations. The number of participants in this study was relatively small, and the patients we employed were mainly patients with major depression. In order to find an effective characterization of depression, it is necessary to further consider including patients with different severities of depression for research, and explore the relationship between depression scale indicators and EEG characteristics. Moreover, only six EEG channels were included in this study; higher density EEG recording should lead to more accurate results. Due to the spatial limitations of the cortical EEG, in order to further accurately explore the study of sleep brain function in patients with depression, high spatial resolution monitoring methods will be considered in future work. It is worth noting that our research focuses on the differences in different sleep stages, and the time dynamics of the characteristics throughout the night required further research. We also explored the possible relationship between information flow and brain function, and more experimental exploration and verification are needed in the future. The clinical application value of the features extracted in this work will be further explored in future work. Since depression is also accompanied by changes in heart rate variability (Koch et al., 2019), respiration pattern variability (Zamoscik et al., 2018), and parasympathetic activity (An et al., 2020), research on the multi-physiological system (central nervous system–cardiorespiratory interaction) of depression will be further investigated. Furthermore, although different information theory methods may reach various conclusions, we believe these methods have their respective advantages in tapping different physiological phenomena, and the relations and differences between them need more research.

Overall, the application of dSPTE reveals the information transmission during sleep. Our results mainly include the right-to-left and posterior-to-anterior superiority in information transmission during sleep, and such directional bias of information flow was attenuated in depressed patients. Our findings may provide new insights for understanding the impact of sleep abnormalities on cognitive function and neuropsychiatric deficits in depressed patients, and provide new clues for the quantitative characterization of depression.

## Data Availability Statement

The datasets presented in this article are not readily available because privacy or ethical restrictions. Requests to access the datasets should be directed to YL, luoyuc@163.com.

## Ethics Statement

The studies involving human participants were reviewed and approved by the Ethics Committee of the Guangdong 999 Brain Hospital. Written informed consent to participate in this study was provided by the participants’ legal guardian/next of kin.

## Author Contributions

JKL contributed to the design of the study and wrote the first draft of the manuscript. JKL and YL collected the data. JKL, MZ, and JZ performed the statistical analysis. YL, JXL, JW, and XG interpreted the results. All authors provided comments, contributed to manuscript revision, and approved the submitted version.

## Conflict of Interest

The authors declare that this study received funding from De Rucci Healthy Sleep Co., Ltd. The funder was not involved in the study design, collection, analysis, interpretation of data, the writing of this article, or the decision to submit it for publication.

## Publisher’s Note

All claims expressed in this article are solely those of the authors and do not necessarily represent those of their affiliated organizations, or those of the publisher, the editors and the reviewers. Any product that may be evaluated in this article, or claim that may be made by its manufacturer, is not guaranteed or endorsed by the publisher.

## Supplementary Material

Supplementary Figure 1 Differences in AP and LR within groups across sleep stages. Black asterisks denote the significant difference between two stages. **p* < 0.05, ***p* < 0.005, and ****p* < 0.001 (Bonferroni correction). The red dotted line is the baseline of AP (AP = 1) /LR (LR = 0).
