# Supplementary figures and images for: Sleep-Dependent Anomalous Cortical Information Interaction in Patients With Depression
Source: Front Neurosci. 2022 Jan 6;15:736426. doi: 10.3389/fnins.2021.736426 (PMC8772413; doi:10.3389/fnins.2021.736426)

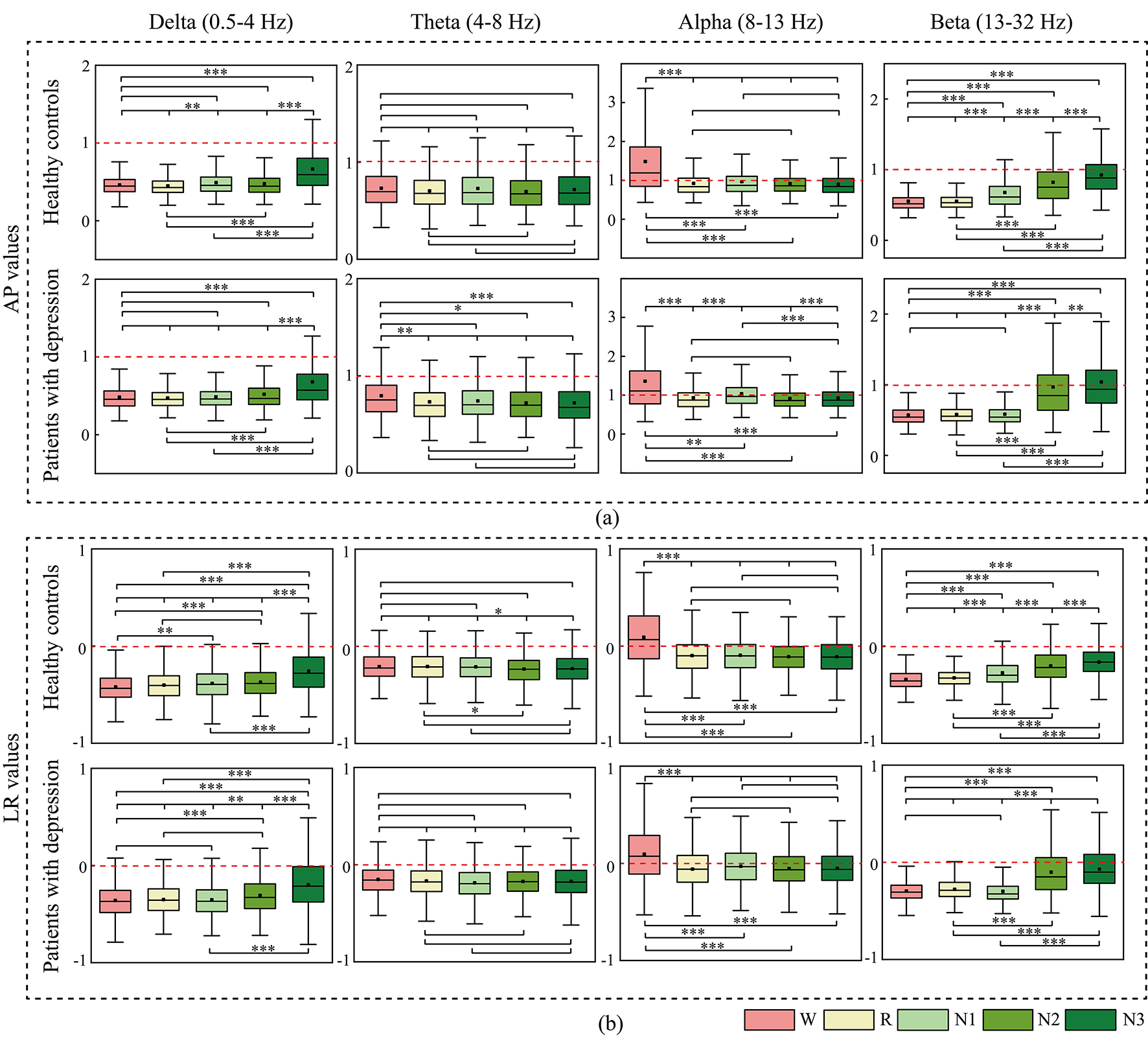

Supplement: Supplementary Figure 1 — Differences in AP and LR within groups across sleep stages. Black asterisks denote the significant difference between two stages. *p < 0.05, **p < 0.005, and ***p < 0.001 (Bonferroni correction). The red dotted line is the baseline of AP (AP = 1) /LR (LR = 0). [file Image_1.TIF]
