# Supplementary material for: Sleep-Dependent Anomalous Cortical Information Interaction in Patients With Depression
Source: Front Neurosci. 2022 Jan 6;15:736426. doi: 10.3389/fnins.2021.736426 (PMC8772413; doi:10.3389/fnins.2021.736426)
Supplement: Supplementary file 2 [file Table_1.DOCX]

**Supplementary Table S1.** Comparison of dSPTE values in different sleep stages

| Groups | Bands | Sleep Stages | | | | | *p* |
| --- | --- | --- | --- | --- | --- | --- | --- |
|  |  | W | R | N1 | N2 | N3 |  |
| Health controls | Delta | 0.12±0.01 | 0.12±0.01 | 0.12±0.01 | 0.12±0.01 | 0.11±0.01 | <0.001 |
|  | Theta | 0.31±0.03 | 0.29±0.02 | 0.30±0.03 | 0.29±0.02 | 0.29±0.02 | <0.001 |
|  | Alpha | 0.43±0.08 | 0.39±0.04 | 0.42±0.05 | 0.43±0.05 | 0.42±0.04 | <0.001 |
|  | Beta | 0.79±0.06 | 0.77±0.04 | 0.77±0.05 | 0.75±0.05 | 0.74±0.06 | <0.001 |
| Patients | Delta | 0.12±0.01 | 0.12±0.01 | 0.12±0.01 | 0.12±0.01 | 0.11±0.01 | <0.001 |
|  | Theta | 0.32±0.04 | 0.29±0.03 | 0.29±0.03 | 0.29±0.02 | 0.29±0.02 | <0.001 |
|  | Alpha | 0.43±0.09 | 0.40±0.05 | 0.42±0.05 | 0.45±0.06 | 0.44±0.05 | <0.001 |
|  | Beta | 0.79±0.05 | 0.78±0.04 | 0.80±0.05 | 0.78±0.08 | 0.76±0.07 | <0.001 |

*Note: All data are presented as mean ± standard deviation, The comparison of sleep stages was assessed with the Friedman test*
