# Supplementary material for: Sleep-Dependent Anomalous Cortical Information Interaction in Patients With Depression
Source: Front Neurosci. 2022 Jan 6;15:736426. doi: 10.3389/fnins.2021.736426 (PMC8772413; doi:10.3389/fnins.2021.736426)
Supplement: Supplementary file 3 [file Table_2.DOCX]

**Supplementary Table S2.** Comparison of AP and LR values in different sleep stages

| Features | Groups | Bands | Sleep stages | | | | | *p* |
| --- | --- | --- | --- | --- | --- | --- | --- | --- |
|  |  |  | W | R | N1 | N2 | N3 |  |
| AP | Health controls | Delta | 0.46±0.12 | 0.44±0.12 | 0.49±0.17 | 0.47±0.15 | 0.66±0.31 | <0.001 |
|  |  | Theta | 0.73±0.21 | 0.70±0.19 | 0.72±0.22 | 0.69±0.18 | 0.71±0.20 | 0.275 |
|  |  | Alpha | 1.48±0.91 | 0.92±0.33 | 0.96±0.40 | 0.91±0.27 | 0.89±0.28 | <0.001 |
|  |  | Beta | 0.55±0.16 | 0.55±0.12 | 0.67±0.24 | 0.82±0.30 | 0.92±0.28 | <0.001 |
|  | Patients | Delta | 0.48±0.15 | 0.47±0.13 | 0.48±0.15 | 0.52±0.20 | 0.68±0.41 | <0.001 |
|  |  | Theta | 0.79±0.24 | 0.73±0.21 | 0.74±0.21 | 0.72±0.19 | 0.72±0.21 | <0.001 |
|  |  | Alpha | 1.36±0.93 | 0.93±0.32 | 1.04±0.36 | 0.91±0.27 | 0.93±0.30 | <0.001 |
|  |  | Beta | 0.57±0.14 | 0.57±0.13 | 0.58±0.16 | 0.96±0.49 | 1.03±0.46 | <0.001 |
| LR | Health controls | Delta | - 0.42±0.14 | - 0.40±0.15 | - 0.38±0.17 | - 0.37±0.17 | - 0.25±0.22 | <0.001 |
|  |  | Theta | - 0.20±0.14 | - 0.20±0.14 | - 0.21±0.14 | - 0.23±0.13 | - 0.22±0.14 | 0.003 |
|  |  | Alpha | 0.09±0.28 | - 0.09±0.18 | - 0.09±0.18 | - 0.10±0.16 | - 0.11±0.17 | <0.001 |
|  |  | Beta | - 0.34±0.11 | - 0.32±0.08 | - 0.27±0.13 | - 0.20±0.16 | - 0.16±0.15 | <0.001 |
| LR | Patients | Delta | - 0.35±0.17 | - 0.35±0.16 | - 0.35±0.17 | - 0.30±0.18 | - 0.19±0.25 | <0.001 |
|  |  | Theta | - 0.15±0.15 | - 0.16±0.15 | - 0.18±0.16 | - 0.17±0.14 | - 0.17±0.15 | 0.079 |
|  |  | Alpha | 0.09±0.28 | - 0.05±0.19 | - 0.03±0.19 | - 0.05±0.17 | - 0.05±0.19 | <0.001 |
|  |  | Beta | - 0.29±0.11 | - 0.27±0.10 | - 0.29±0.12 | - 0.10±0.23 | - 0.07±0.21 | <0.001 |

*Note: All data are presented as mean ± standard deviation, The comparison of sleep stages was assessed with the Friedman test.*
